# A Fusion Protein Based on the Second Subunit of Hemagglutinin of Influenza A/H2N2 Viruses Provides Cross Immunity

**Published:** 2016

**Authors:** L. A. Stepanova, M. V. Sergeeva, M. A. Shuklina, A. A. Shaldzhyan, M. V. Potapchuk, A. V. Korotkov, L. M. Tsybalova

**Affiliations:** Research Institute of Influenza, Prof. Popova Str. 15/17, 197376, St. Petersburg, Russia

**Keywords:** vaccine, influenza, HA2, recombinant protein, flagellin

## Abstract

Conserved fragments of the second subunit of hemagglutinin (HA2) are of great interest for the design of vaccine constructs that can provide protective immunity against influenza A viruses of different subtypes. A recombinant fusion protein, FlgMH, was constructed on the basis of flagellin and a highly conserved HA2 fragment (35–107) of influenza viruses of the subtype A/H2N2, containing B cell, CD4+ T cell, and CD8+ T cell epitopes. The native conformation of the HA2 fragment was partially preserved upon its attachment to the C-terminus of flagellin within the recombinant fusion protein FlgMH. FlgMH was shown to stimulate a mixed Th1/Th2 response of cross-reactive antibodies, which bind to influenza viruses of the first phylogenetic group (H1, H2, H5), to the target sequence as well as the induction of specific cytotoxic T cells (CD3+CD8+IFNγ+). Immunization with the recombinant protein protected animals from a lethal influenza infection. The developed FlgMH protein is a promising agent that may be included in an influenza vaccine with a wide spectrum of action which will be able to stimulate the T and B cell immune responses.

## INTRODUCTION

Virus-neutralizing antibodies prevent infection by blocking the attachment of the influenza virus to the cell surface. These antibodies are mainly targeted at immunodominant epitopes of the first highly variable subunit of hemagglutinin (HA1). The narrow specificity of neutralizing antibodies makes existing vaccines ineffective against circulating influenza virus variants and against emerging viruses with a pandemic potential. T cell immunity significantly contributes to viral clearance and facilitates a mild infection. Therefore, vaccines inducing not only humoral, but also T cell responses are desirable for a better control of an influenza infection. The development of vaccines based on conserved antigens that enhance both the humoral and cellular responses is a universal strategy to control epidemics or pandemics.

Influenza virus hemagglutinin is a polypeptide synthesized as a precursor (HA0) that is trimerized in the endoplasmic reticulum and transported via the Golgi apparatus to the cell surface. Hemagglutinin (HA0) is post-translationally cleaved by host proteases into two subunits, HA1 and HA2, that remain linked by one disulfide bridge [1]. Unlike HA1, the second subunit of hemagglutinin (HA2) has a relatively conserved sequence among viral strains and is responsible for the fusion of the viral and cell membranes in endosomes, thereby ensuring entry of the ribonucleic complex into the cytoplasm [2].

Immunization with traditional vaccines and natural influenza infection do not lead to the formation of a significant amount of anti-HA2 antibodies, which is associated with the low immunogenicity of the HA2 stalk region in the presence of immunodominant receptor- binding regions of HA1 [3]. However, a number of monoclonal antibodies (mouse, human) have been recently isolated that interact with epitopes localized in the HA stalk. These are cross-reactive antibodies that neutralize influenza virus subtypes within the phylogenetic group, thereby providing a wide range of protection [4-11].

Monoclonal antibodies specific to the 1–38 (CF2) and 125–175 (FE1) HA2 regions are capable of *in vitro *inhibiting the fusogenic activity of the influenza A virus. Intravenous administration of CF2 and FE1 monoclonal antibodies 2 h before infection of Balb/c mice with homologous and heterologous influenza viruses A/H3N2 at the 1LD_50_ dose provided 100% survival of animals [6]. The monoclonal antibody CR6261 specific to the hydrophobic pocket of the HA2 stalk inhibited pH-induced conformational change in HA of the A/H1N1 and A/H5N1 influenza viruses and had a neutralizing activity [4, 5]. The monoclonal antibody CR6261 was shown to interact predominantly with the HA2 α-helix, as well as with the adjacent amino acid residues of HA1 and HA2. Most amino acids in the small α-helix of HA2, which interact with CR6261, are identical by more than 99% (differences in 1–5 amino acid residues) within influenza virus subtypes of the first phylogenetic group. The monoclonal antibody CR6261 prevented a transition of HA to the post-fusogenic conformation at low pHs; i.e., it neutralized the virus through stabilization of the pre-fusogenic state and prevention of the pH-dependent fusion of the viral and cell membranes. The anti-H3N2 monoclonal antibody 12D1that interacts with the large, highly conserved α-helix (residues 76–106) of HA2 has a neutralizing ability and bounds to the A/H3N2 influenza viruses that circulated from 1968 to 2003 [8].

In recent years, a number of potential vaccines on the basis of HA2 of influenza A viruses from the phylogenetic group II have been developed [12-14]. The immunogenicity and efficacy of the vaccines in protecting from infection with lethal doses of homologous and heterologous viruses of one phylogenetic group have been demonstrated. The design of the antigen inducing an immune response to HA2 conserved epitopes may provide the basis for a broad-spectrum vaccine possessing prophylactic and therapeutic efficacy.

The aim of this work was to model and generate a recombinant fusion protein comprising the promising T and B cell epitopes of HA2 of the influenza viruses A/ H2N2 and to study the immunogenicity and protective action of the protein. Flagellin, a mucosal adjuvant that enhances the immune response to attached antigens, was chosen as the basis for the fusion protein.

## MATERIALS AND METHODS

**Selection of a conserved HA2 region of the influenza A/H2N2 viruses**

The search for amino acid sequences for analysis was carried out in the GenBank database; alignments were performed using the Vector NTI v10.0 software (Invitrogen, USA). The search for possible T cell epitopes was conducted using the NetCTLpan 1.1 Server [15] and default search parameters. The search for experimental B and CD4+ T cell epitopes homologous to HA2 fragments was performed in the Immune Epitope Database [16]. The three-dimensional structure of proteins was visualized using a Chimera 1.5.3 program [17]. A Phyre2 open web resource was used for primary sequence homology simulation of the three-dimensional protein structure [18].

**Construction of expression vectors and generation of *Escherichia coli* host expression**

The nucleotide sequence encoding the recombinant fusion protein FlgMH was optimized for expression in *E. coli*, synthesized, and inserted into the pQE30 vector at the BamHI and HindIII restriction sites. To generate Flg (flagellin) and MH (hemagglutinin fragment) proteins, the appropriate nucleotide sequences were amplified using primers carrying the terminal restriction sites BamHI and HindIII and then inserted into the multicloning site of the pQE30 vector. DLT1270* E. coli *cells were transformed with the pQE30/Flg- MH, pQE30/MH, and pQE30/Flg plasmids to generate strains producing recombinant proteins. The DLT1270 strain, a derivative of the DH10B strain [19], contained the *lacI *lactose operon repressor gene integrated into the chromosome.

**Isolation and purification of recombinant proteins**

DLT1270 *E. coli *strains transformed with the pQE30/ FlgMH, pQE30/Flg, and pQE30/MH vectors were cultured in LB medium supplemented with ampicillin. Expression was induced by adding 1 mM IPTG. Cells were treated with lysozyme, and recombinant proteins were purified from the cell lysate using metal affinity chromatography on a Ni-sorbent.

**Electrophoresis and immunoblotting**

Polyacrylamide gel electrophoresis (PAGE) under denaturing conditions was carried out according to the Laemmli method [20]. Samples were mixed with a loading buffer containing β-mercaptoethanol, boiled for 7 min, and loaded into 8–16% gradient PAG. Electrophoresis was performed at 10–12 mA for 1.5 h. The gel was fixed in 10% acetic acid and then stained with Coomassie G-250 for 18 h.

Horizontal transfer of the proteins from polyacrylamide gel to a nitrocellulose membrane (BioRad, USA) was performed in TB-buffer (0.03 M glycine, 0.04 M Tris, 0.037% sodium dodecyl sulfate, 20% ethanol) using a Mini Trans-Blot cell system (BioRad, USA) in a chilled chamber at +4°C and a constant current of 200 mA for 1.5 h. The membrane was then blocked in a 3% bovine serum albumin (BSA) solution (Amresco, EU) in phosphate-buffered saline (PBS) at room temperature overnight. The membrane was incubated with primary antibodies diluted in PBS with 0.1% Tween 20 (PBST) and 3% BSA at room temperature for 1 h and then washed in PBST. Flagellin was stained with rabbit polyclonal antibodies (Abcam, UK) at a 1 : 16,000 dilution. A hemagglutinin fragment was stained with cross-specific serum, which was produced by triple sequential immunization of mice with sublethal doses of influenza A viruses of the phylogenetic group I (H2, H5, and H1pdm), at a 1 : 2,000 dilution. Proteins were detected by staining the membrane with secondary antibodies labeled with horseradish peroxidase (goat anti-rabbit IgG or goat anti-mouse IgG, Invitrogen, USA) at a 1 : 2,000 dilution at room temperature for 1 h and then incubated with a tetramethylbenzidine (TMB) immunoblot substrate solution (Invitrogen, USA) for 15 min.

**Immunization of mice**

The FlgMH recombinant protein immunogenicity was studied in linear mice Balb/c and C57Bl/6 (females, age of 6–8 weeks, weight of 18–20 g) received from the Stolbovaya mouse farm of the State Scientific Center of Biomedical Technologies of the Russian Academy of Medical Sciences. The animals were kept at the vivarium of the Research Institute of Influenza in accordance with working regulations. Mice (16 animals) were intranasally immunized with the recombinant protein Flg- MH (after inhalation anesthesia with 2–3% isoflurane, 30% O_2_, 70% N_2_O) at a dose of 10 μg/mouse in a volume of 50 μL three times with a two-week interval. Control mice (16 animals) were intranasally administered the recombinant protein Flg at a dose of 10 μg/mouse three times with a two-week interval.

**Collection of sera and BAL**

Blood samples were obtained from five mice from the experimental and control groups 2 weeks after the last immunization, following euthanasia in a CO_2_-chamber (Vet Tech Solutions, UK). To obtain serum, blood samples were incubated at 37 °C for 30 min. After blood clot formation, the samples were placed on ice and cooled for 1 h, followed by centrifugation at 400*g *for 15 min. Serum aliquots (30 μL) from five mice of each group were frozen at –20°C.

Bronchoalveolar lavages (BALs) were obtained from five mice of each group 2 weeks after the last immunization of the animals, following euthanasia in the CO_2_-chamber. An animal’s corpse was fixed on the operating table, with the belly up. A skin incision was made along the midline, starting from the mandible. A catheter was inserted into the lower portion of the trachea to a depth of 3– mm towards the lungs. The bronchi and lungs were washed twice with 1 mL of PBS. BAL was centrifuged at 400*g *for 15 min. The supernatant was aliquoted and frozen at –20°C.

**Collection of mouse splenocytes**

Mouse splenocytes were prepared according to the BD PharmingenTM protocol. Mice from the experimental and control groups (three mice from each group) were euthanized using the CO_2_-chamber on the 14th day after the last immunization. Mouse spleens were removed aseptically, homogenized using a Medimachine (BD Biosciences, USA), and purified from cell debris by filtration through a syringe filter with a 70 μm pore size (Syringe Filcons, BD Biosciences, USA). Erythrocytes were lysed with ACK lysing buffer (0.15 M NH_4_Cl, 1.0 M KHCO_3_, 0.1 mM Na_2_EDTA, pH 7.2–7.4); splenocytes were washed with a complete RPMI- 1640 medium with 10% FBS, 2 mM *L*-glutamine, 100 U/mL penicillin, and 100 μg/mL streptomycin. Cell viability was assessed by staining with a 0.4% trypan blue solution. The cell concentration was adjusted to 2 × 10^6^ cells/mL.

**Synthetic peptides**

The immunogenicity of recombinant proteins was evaluated using the following synthetic peptides synthesized by Verta (Russia):

G-47 (24 amino acids): AADKESTQKAFDGITNKVNSVIEK, the small α-helix (35–58) of HA2; G-48 (15 amino acids): MNTQFEAVGKEFSNL, an unfolded “linker” segment (59–2) of HA2 in native hemagglutinin; G-49 (34 amino acids): ERRLENLNKKMEDGFLDVWTYNAELLVLMENERT, a fragment of the large α-helix (73–07) of HA2.

**Enzyme-linked immunosorbent assay**

ELISA was performed according to a conventional method. 96-well plates with a high sorption capacity (Greiner, Germany) were coated with the recombinant protein FlgMH at a concentration of 5 μg/mL or purified viruses A/Singapore/1/57 (H2N2), A/PR/8/34 (H1N1), A/Aichi/1/68 (H3N2), A/Kurgan/05/2005/ RG (H5N1) at a concentration of 2 μg/mL; sorption was performed in PBS, pH 7.2, at 4°C overnight. After virus sorption, parts of the plates were immersed in citrate buffer (pH 5.0) for 30 min and then washed once by PBS. Plates were treated with blocking buffer (0.01 M PBS with 5% FBS, pH 7.2) at room temperature for 1 h and washed 3 times with PBST. Plate wells were filled with 100 μL of two-fold dilutions of sera (starting with 1 : 400) in blocking buffer and incubated at room temperature for 1 h. Polyclonal HRPO-labelled goat anti-mouse IgG, IgG1, IgG2a, IgG2b, IgG3, and IgA antibodies (Abcam, UK) a 1 : 20,000 dilution were used. TMB (BD Bioscience, USA) was used as a substrate; the incubation time was 15 min. The optical density (OD) was measured using the i-Mark microplate reader (Bio-Rad) at a wavelength of 450 nm. The maximal serum dilution that had an optical density at least 2 times higher than the double mean value of the blank was taken as the titer.

**Flow cytometry**

Multiparameter flow cytometry was performed according to the BD PharmingenTM protocol. The ability of the G-47, G-48, and G-49 synthetic peptides and the influenza virus A/Singapore/1/57 (H2N2) to activate the production of IFN-γ by specific CD8+ T cells in the spleen was determined. Splenocytes of Balb/c and C57Bl/6 mice were obtained on the 14th day after the last immunization; 2 × 10^6^ of splenocytes from mice of the experimental and control groups were stimulated (at 37°C for 6 h) with 10 μg of the G-47, G-48, and G-49 peptides or 1 μg of the virus A/Singapore/1/57 (H2N2) in the presence of brefeldin A (1 μg/mL) (BD Bioscience, USA). Cells were washed, and Fc-receptors were blocked by CD16/CD32 antibodies (Mouse BD Fc Block, BD Pharmingen, USA) and stained with anti- CD3a-FITC and anti-CD8-PerCP (BD Pharmingen, USA), at 4°C for 30 min. Then, the cells were permeabilized in accordance with the Cytofix/Cytoperm Plus kit protocol (BD Bioscience, USA) and stained with anti- IFN-γ-PE antibodies (BD Pharmingen, USA). The fluorescence intensity was measured on a BD FACS Canto II flow cytometer (Becton Dickinson, USA). Results were analyzed using the BD FACSDiva v6.1.3 (BD Bioscience, USA) software.

**Viruses and infection of mice**

We used strains received from the Collection of influenza and ARD viruses of the Laboratory of Evolutionary Variability of Influenza Viruses of the Research Institute of Influenza: A/Singapore/1/57 (H2N2), A/PR/8/34 (H1N1), A/Aichi/2/68 (H3N2), and A/Kurgan/05/2005/RG (H5N1). In experiments with lethal infection, we used a variant of the influenza A/Singapore/1/57 (H2N2) virus adapted to reproduce in the mouse lungs that was produced at the Laboratory of Influenza Vaccines of the Research Institute of Influenza. Viruses were accumulated in 10- to 12-dayold chicken embryos and purified by ultracentrifugation in a sucrose gradient.

Immunized mice (11 mice each from the experimental and control groups) were infected with the mouse-adapted influenza virus A/Singapore/1/57 (H2N2) at a 2LD_50_ dose. The virus was administered intranasally in a volume of 50 μL per mouse after inhalational anesthesia (2–% isoflurane, 30% O_2_, 70% N_2_O). After infection, the animals were subjected to everyday monitoring. The protective effect of FlgMH was evaluated based on two parameters: dynamics of body weight loss and survival of mice after infection.

**Statistical processing**

Statistical data processing was carried out using the GraphPad Prizm v5.1 program. The statistical significance of antibody titer differences was evaluated using the nonparametric Mann-Whitney test. Comparison of survival rates was performed using the Mantel-Cox test. The differences were considered significant at *p * < 0.05.

## RESULTS AND DISCUSSION

**Construction of the MH-fragment of the HA2 consensus sequence of the influenza virus A/H2N2**

**Fig. 1:**
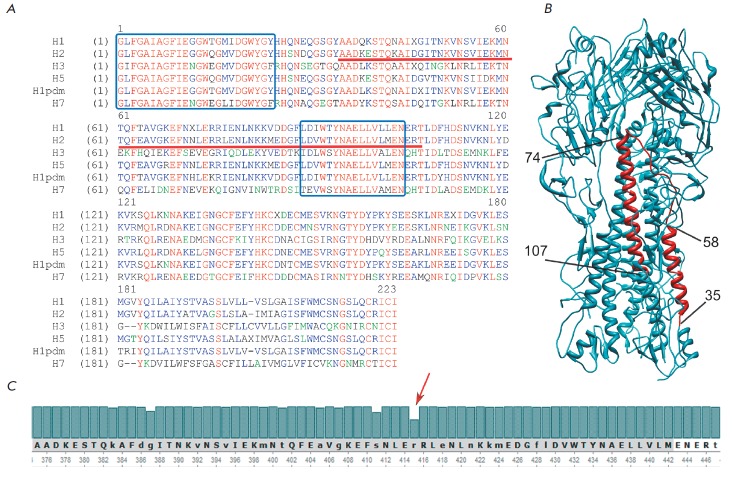
A – alignment of HA2 consensus sequences of influenza A viruses of different subtypes. Two highly conserved fragments with sequence identities of 78.3 and 62.5%, respectively, are shown in blue boxes. A fragment selected for inclusion in the fusion protein is underlined. B – the three-dimensional structure of the hemagglutinin molecule (trimer, a model 3WR7 from the Protein Data Bank). The HA2 fragment (35–107) of one of the monomers is shown in red. Main fragments: the small α-helix (35–58); an unstructured region (59–73); a fragment of the large α-helix containing a highly conserved fragment (74–107). C – amino acid frequency plot of a HA2(35-107) fragment of human influenza A/H2N2 hemagglutinin (in HA0 numbering), the less conserved residue (Arg416 – 58.3%) is indicated by a red arrow.

**Fig. 2:**
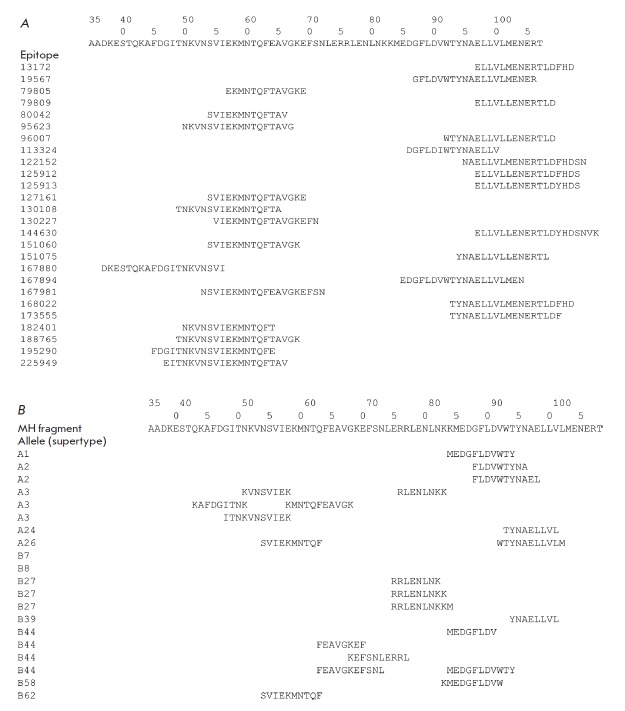
A – experimental B and CD4+ T cell epitopes homologous to the HA2 consensus sequence fragment (35–107) at least by 90%; based on the search in the IEDB database. B – potential CD8+ T cell epitopes included in the HA2 fragment (35–107) for a representative set of HLA alleles; the result of an analysis using the NetCTLpan1.1 Server [19].

Two highly conserved fragments, (1–24) and (89– 104), were found in the alignment of consensus sequences of HA2 (*Fig. 1A*). The identity of the first fragment was 78.3%, but the fragment contained a highly hydrophobic HA fusion peptide prone to aggregation. Although the second fragment was less conserved (62.5%), the majority of its amino acid substitutions were not associated with changes in the physicochemical properties of side chains, which gives hope for a small change in the ability to be presented in certain HLA alleles. Furthermore, the conserved sequence YNAELLVL, which is a part of this fragment, was found in most B and CD4+ T epitopes in HA2 (*Fig. 2A*). The H3 and H7 sequences (phylogenetic group II) were the most differentianted from the consensus sequence; their exclusion increased the fragment identity to 87.5%. A fragment of the HA2 consensus sequence of human influenza viruses A/H2N2 (phylogenetic group I) was chosen for construction of the target recombinant fusion protein.

The fusion protein included the conserved HA2 fragment (89–104), as well as the small α-helix (35–58) of HA2, which was exposed to the trimer surface and was potentially available for binding to antibodies (*Fig. 1B*). To preserve the tertiary structure, the fusion protein FlgMH included a continuous HA2 fragment (35–107).

The HA2 fragment (35–107) of human H2 influenza viruses has the highest amino acid composition homogeneity. The most variable amino acid (occurrence of 58.3%) was arginine (R) at position 75 (position 415 in the HA0 numbering), with lysine (K) occurring in this position in 41.7% of the cases. Both amino acids were positively charged, but the arginine side chain was larger; therefore, it is preferable to provoke a humoral immune response (*Fig. 1B*).

Furthermore, the HA2 sequence (35–107) contained fragments homologous to experimental B and CD4+ T cell epitopes present in the IEDB database (*Fig. 2A*). The theoretical search revealed the presence of multiple potential CD8+ T cell epitopes in the HA2 fragment (35–107) for a representative set of alleles (*Fig. 2B*).

**Design of the recombinant fusion protein FlgMH**

**Fig. 3:**
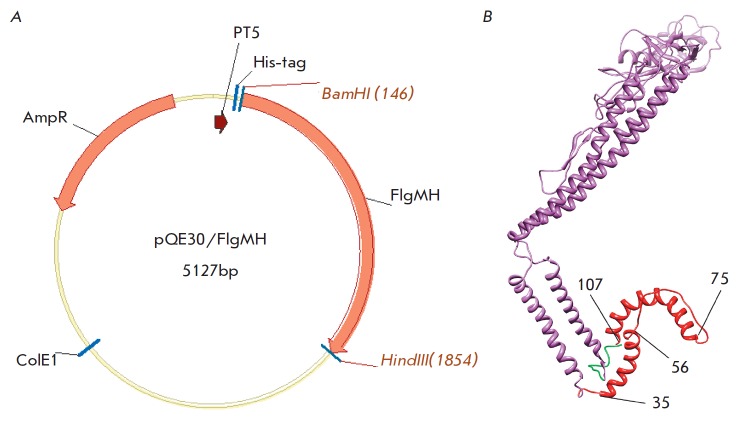
A – map of a plasmid encoding the protein FlgMH. BamHI and HindIII are cloning sites of the gene encoding the fusion protein FlgMH into the vector pQE30. ColE1 – origin of replication; PT5 – the T5 promoter; AmpR – the β-lactamase gene, an ampicillin resistance marker; His-tag – a N-terminal histidine tag. B – a theoretical model of the three-dimensional structure of a monomer of the fusion protein FlgMH: the HA2 fragment (35–107) is shown in red; flagellin is shown in violet; the histidine tag is shown in green.

The chimeric protein was constructed using the commercial plasmid pQE30 containing a start codon and a histidine tag before the cloning site. The fusion protein FlgMH included the full-length sequence of flagellin (FliC), lacking a start codon, and the target MH sequence that were encoded by a single reading frame with the histidine tag (*Fig. 3A*). Therefore, the recombinant protein FlgMH consisted of flagellin, with the histidine tag at the N-terminus and the HA2 consensus sequence (35–107) of human influenza A/H2N2 viruses at the C-terminus. Homologous modeling of the three-dimensional FlgMH structure demonstrated preservation of the α-helix structure in MH regions corresponding to HA2 fragments (38–56) and (75–107) (*Fig. 3B*), suggesting that most of the native structure was preserved, and the fusion protein would stimulate the formation of antibodies, in particular to structural epitopes typical of native HA.

**Production and purification of recombinant proteins**

**Fig. 4:**
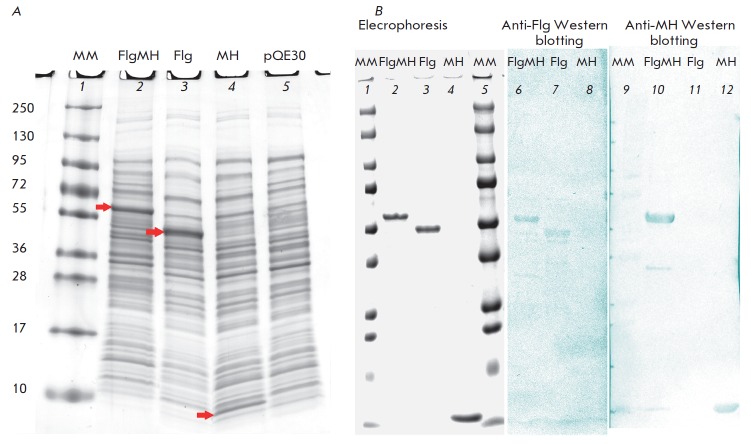
A – expression of recombinant proteins in E. coli cells. 1 – molecular weight markers (Fermentas, EU) denoted in kDa; 2–4 – lysates of cells transformed with the plasmid pQE30 carrying an indicated insert; 5 – a lysate of cells transformed with the vector pQE30 without an insert. Bands of recombinant proteins are indicated by arrows. B – recombinant proteins FlgMH, Flg, and MH after chromatographic purification on a Ni-sorbent. Results of electrophoresis and Western blot using polyclonal antibodies to flagellin (6–8) and antiserum to a fragment MH (9–12) are presented.

The nucleotide sequences encoding the fusion protein FlgMH, as well as its components Flg and MH, were cloned into the pQE30 vector and expressed in the DLT1270 *E. coli *strain (*Fig. 4A*). The theoretical molecular weights of the proteins were as follows: FlgMH (61.3 kDa), Flg (52.9 kDa), and MH (9.8 kDa), which coincided with their electrophoretic mobility in polyacrylamide gel (*Fig. 4B*). The Flg and FlgMH proteins were soluble in PBS, unlike the MH protein that accumulated in inclusion bodies and dissolved only in 2 M urea. In western blotting, the purified proteins FlgMH and Flg interacted with anti-flagellin rabbit polyclonal serum and the proteins FlgMH and MH interacted with mouse cross-specific serum against HA of influenza viruses of phylogenetic group I (*Fig. 4B*).

**Immunogenicity and protective ability of the recombinant protein FlgMH**

The HA2 consensus sequence (35–107) included in the FlgMH protein contains B, CD4+, and CD8+ T cell epitopes. Thus, we assessed the ability of the recombinant protein FlgMH to stimulate both the B and T cell immune responses.

**Fig. 5:**
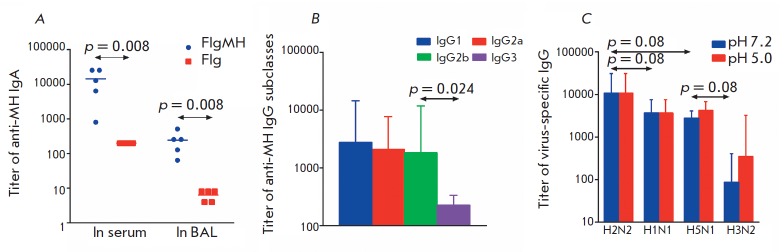
Antibody titers in mice of the experimental and control groups 2 weeks after triple immunization. A – titers of IgA antibodies, in serum and BAL, to the target antigen MH. B – GMT of IgG subclasses to the target antigen MH in mice of the experimental group. C – GMT of serum IgG to influenza viruses A/PR/8/34 (H1N1), A/Singapore/1/57 (H2N2), A/Aichi/2/68 (H3N2), and A/Kurgan/05/2005 (H5N1). The Mann-Whitney test was used to calculate the p value.

To investigate the ability of the recombinant protein FlgMH to induce the formation of HA2-specific antibodies, Balb/c mice were immunized intranasally three times with the FlgMH protein without the adjuvant; mice of the control group were administered the Flg protein. The intranasal route of antigen administration induces both systemic and local immune responses. Therefore, on day 14 after the last immunization, we determined the serum and BAL levels of IgA to the target antigen and the serum titers of IgG to influenza viruses of the first and second phylogenetic groups and evaluated the profile of IgG subclasses (IgG1, IgG2a, IgG2b, IgG3). The local response is associated with secretory sIgA, whose multimer form has effective antiviral activity, inhibiting viral replication [21, 22]. Intranasal immunization of mice with the recombinant protein FlgMH stimulated a high level of anti-HA2 IgA in the serum and BAL of the immunized animals (*Fig. 5A*).

The type of immune response by flagellin and flagellin- based recombinant proteins is known to be determined by the flagellin shape. Soluble flagellin (monomeric and polymeric) induces an immune response specific to flagellin and the co-administered target antigen, with a strong predominance of the Th2-type response [23-29]. At the same time, membrane-anchored flagellin induces primarily a Th1-type immune response [24, 28]. On the other hand, the type of an immune response to the target antigen was shown to be also dependent on the flagellin-fused antigen [23]. As shown in *Fig. 5B*, immunization with the soluble recombinant protein FlgMH led to induction of almost equal levels of HA2-specific antibodies IgG1 (type Th2 response) and IgG2a and IgG2b (type Th1 response): IgG1 – 40.0%, IgG2a – 30.3%, IgG2b – 26.4%, and IgG3 – 3.3%. The absence of significant differences in IgG subclasses (IgG1, IgG2a, IgG2b) for the target antigen after immunization of mice with the recombinant protein FlgMH suggests a mixed Th1 and Th2 immune response.

The selected HA2 consensus sequence (35–107) is quite conserved in influenza viruses of the first phylogenetic group (87.5% homology); therefore, it was important to evaluate the formation of cross-reactive antibodies after immunization of mice with the Flg- MH protein. According to the ELISA, FlgMH-induced HA2-specific IgGs bound not only to the influenza virus of the A/H2N2 subtype (geometric mean titer, GMT = 12,800), but also to other influenza A virus subtypes from the first phylogenetic groups: H1 (GMT = 4,160) and H5 (GMT = 2,880) (*Fig. 5B*). However, titers of antibodies to influenza viruses of the H5 and H1 subtypes were significantly lower than that to the H2 subtype (*p* < 0.05, Mann-Whitney test). In addition, induced antibodies bound to hemagglutinin in native conformation (pH 7.2) and to an acidic form of hemagglutinin (pH 5.0) with the same affinity (*Fig. 5B*), indicating the accessibility of the target HA2 sequence on the virion surface for antibodies.

**Fig. 6:**
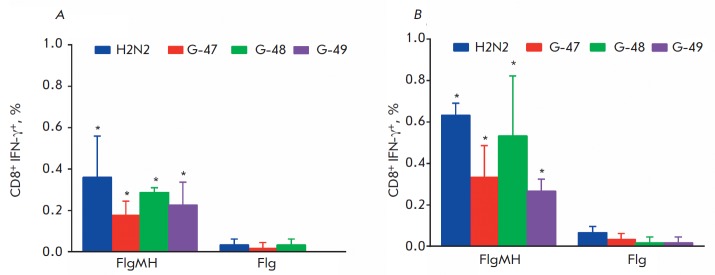
IFNγ production by spleen CD8+ T-cells after re-stimulation with the G-47, G-48, and G-49 peptides and the influenza virus A/Singapore/1/57 (H2N2) in mice C57Bl/6 (A) and Balb/c (B). The spleens were taken from three mice each of the experimental and control groups 14 days after triple immunization. * – the difference from control p < 0.05 (Mann-Whitney test).

The ability of the recombinant protein FlgMH to induce a cellular response was determined based on the production of IFN-γ by spleen CD3+CD8+ T cells after re-stimulation with synthetic peptides (HA2_35–58_, HA2_59– 72_, HA2_73–107_) corresponding to the target HA2 sequence or with a purified influenza virus A/H2N2. The number of activated IFN-γ secreting CD3+ CD8+ T cells both in Balb/c mice (haplotype H-2d) and in C57Bl/6 mice (haplotype H-2b) immunized with the recombinant protein FlgMH was shown to be significantly higher (*p * < 0.05, Mann-Whitney test) than that in mice immunized with the carrier protein flagellin (*Fig. 6A,B*).

Flagellin provides an antigen-specific CD4+ T cell response [30] through activation of TLR5 expressed on CD11c+ cells [31], which leads to a strong humoral response. However, the ability of flagellin to stimulate a specific CD8+ T cell response remains unclear. Several studies have shown that immunization of mice with a fusion protein (flagellin-GFP, flagellin-OVA) stimulates a CD8+ response to the antigen, contrary to immunization with the flagellin-free antigen, only [26, 32]. On the other hand, soluble flagellin fused with an antigen was found to induce predominantly the Th2 response and not to generate antigen-specific CD8+ cells [23-36]. To be presented in the MHC-complex, the antigen undergoes exogenous proteolytic degradation, before which it should be unfolded [33]. Reduction of disulfide bonds in a protein is a key element of the unfolding process [34], and cross-presentation of the antigen, which contains disulfide bridges, by dendritic cells depends on the IFN- γ-induced expression of thiol reductases [35]. Fusion of MHC I-restricted immunogenic epitopes to flagellin was shown [36] to be able to create a pseudo-adjuvant effect that functions via enhanced presentation of the antigen on the cell surface and not to be dependent on TLR5, MyD88, and conserved flagellin fragments. This is related to a more effective processing of the flagellin-fused antigen compared to processing of the antigen in its native state. This means that the antigen, not the TLR5 signal, is a limiting factor in the formation of the CD8+ T cell response. Thus, flagellin may serve as a platform for vaccines containing poorly processed antigens bearing CD8+ epitopes [36].

We found that a soluble form of flagellin fused with the sequence HA2_35–107_ containing CD8+ epitopes stimulates the formation of HA2-specific CD8+ T cells.

**Fig. 7:**
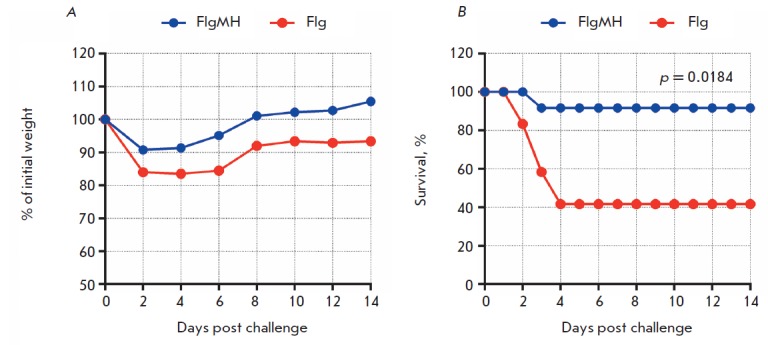
Efficacy of FlgMH immunization. Two weeks post second boost mice were challenged with 2LD50 A/Singapore/ 1/57 (H2N2). Body weight (A) and survival rate (B) were monitored daily during 14 days. The p value was calculated using the Mantel-Cox test.

The ability of the recombinant protein FlgMH to protect mice was demonstrated upon infection of immunized animals with a lethal dose (2LD_50_) of the adapted influenza virus A/Singapore/1/57 (H2N2). Immunization led to differences in body weight dynamics in experimental and control mice: maximum body weight loss was 10% in immunized mice and 16.6% in control mice (*Fig. 7A*). Immunization with the recombinant protein FlgMH protected mice from infection (*Fig. 7B*). The survival rate was 91.0% in the experimental group in contrast to 41% in the control group. The observed differences were statistically significant (*p* = 0.0184, Mantel-Cox test).

## CONCLUSIONS

Conserved fragments of the second HA subunit are of particular interest for the design of vaccine constructs that can provide broad-spectrum immunity against influenza A viruses. The recombinant fusion protein FlgMH based on flagellin and the highly conserved hemagglutinin HA2 fragment (35–107) of influenza viruses of the A/H2N2 subtype includes potential B cell and CD4+ and CD8+ T cell epitopes. It combines the adjuvant activity of flagellin due to its specific binding to TLR5 and the conserved sequences of the hemagglutinin stalk region involved in conformational changes leading to the fusion of the bilayer lipid membranes of the virus and the host cell during a pH-induced fusion reaction. The target sequence including the small α-helix, a fragment of the larger α-helix, and a linker connecting the helices is part of the second hemagglutinin chain and is characterized by very high stability of the amino acid composition within the subtype.

The recombinant protein FlgMH stimulates a mixed Th1/Th2-response to the target sequence, formation of cross-reactive antibodies that bind to influenza viruses of the first phylogenetic group (H1, H2, H5), and induction of specific cytotoxic T cells (CD3+CD8+IFN-γ+). Immunization with the fusion protein protected immunized animals from a lethal influenza infection. The constructed recombinant fusion protein FlgMH is a promising basis for the development of an influenza vaccine with a wide spectrum of action and ability to stimulate the T and B cell immune responses. The cross-protective potential of HA2 fragments can be amplified through optimization of their delivery and increased immunogenicity using ligands of TLR-receptors for effective stimulation of innate immunity and subsequent amplification of the adaptive immune response.

## Abbreviations

HA2: second subunit of hemagglutinin
Flg: flagellin
FlgMH: fusion protein comprising flagellin and a conserved HA2 fragment of influenza viruses A/H2N2
BAL: bronchoalveolar lavage
ELISA: enzyme-linked immunosorbent assay
OD: optical density
GMT: geometric mean titer
TLR5: Toll-like receptor 5
MHC: major histocompatibility complex
